# Hypoxia inhibits colonic uptake of the microbiota-generated forms of vitamin B1 *via* HIF-1α-mediated transcriptional regulation of their transporters

**DOI:** 10.1016/j.jbc.2022.101562

**Published:** 2022-01-05

**Authors:** Subrata Sabui, Kalidas Ramamoorthy, Jose M. Romero, Rita D. Simoes, James M. Fleckenstein, Hamid M. Said

**Affiliations:** 1 Department of Physiology and Biophysics, UCI, Irvine, California, USA; 2 Department of Research, VA Medical Center, Long Beach, California, USA; 3 Department of Medicine, UCI, Irvine, California, USA; 4 Division of Infectious Diseases, Department of Medicine, Washington University School of Medicine, St Louis, Missouri, USA; 5 Department Medicine Service, Veterans Affairs Medical Center, St Louis, Missouri, USA

**Keywords:** hypoxia, vitamin B1 uptake, human differentiated colonoid monolayers, HIF-1α, CREB, cAMP responsive element–binding protein, cTPPT, colonic TPP transporter, DFO, desferrioxamine, Elf-3, E74-like ETS transcription factor 3, GKLF-4, gut-enriched Krüppel-like factor 4, HIF, hypoxia-inducible transcription factor, HRE, hypoxia-responsive element, IBD, inflammatory bowel disease, LDHA, lactate dehydrogenase A, NF-1, neurofibromatosis-1, PGK-1, phosphoglycerate kinase 1, qPCR, quantitative PCR, SP-1, specificity protein 1, THTR-1, thiamin transporter-1, THTR-2, thiamin transporter-2, TPP, thiamin pyrophosphate

## Abstract

Hypoxia exerts profound effects on cell physiology, but its effect on colonic uptake of the microbiota-generated forms of vitamin B1 (*i.e.*, thiamin pyrophosphate [TPP] and free thiamine) has not been described. Here, we used human colonic epithelial NCM460 cells and human differentiated colonoid monolayers as *in vitro* and *ex vivo* models, respectively, and were subjected to either chamber (1% O_2_, 5% CO_2_, and 94% N_2_) or chemically (desferrioxamine; 250 μM)-induced hypoxia followed by determination of different physiological–molecular parameters. We showed that hypoxia causes significant inhibition in TPP and free thiamin uptake by colonic NCM460 cells and colonoid monolayers; it also caused a significant reduction in the expression of TPP (SLC44A4) and free thiamin (SLC19A2 and SLC19A3) transporters and in activity of their gene promoters. Furthermore, hypoxia caused a significant induction in levels of hypoxia-inducible transcription factor (HIF)-1α but not HIF-2α. Knocking down HIF-1α using gene-specific siRNAs in NCM460 cells maintained under hypoxic conditions, on the other hand, led to a significant reversal in the inhibitory effect of hypoxia on TPP and free thiamin uptake as well as on the expression of their transporters. Finally, a marked reduction in level of expression of the nuclear factors cAMP responsive element–binding protein 1 and gut-enriched Krüppel-like factor 4 (required for activity of SLC44A4 and SLC19A2 promoters, respectively) was observed under hypoxic conditions. In summary, hypoxia causes severe inhibition in colonic TPP and free thiamin uptake that is mediated at least in part *via* HIF-1α-mediated transcriptional mechanisms affecting their respective transporters.

Vitamin B1, a water-soluble micronutrient, is essential for normal physiology and health of all human/mammalian cells. In its predominant and biologically active form, that is, thiamin pyrophosphate (TPP), the vitamin plays indispensable roles in oxidative energy metabolism–ATP production and in reduction of cellular oxidative stress. Thus, it is not surprising that deficiency of vitamin B1 at the cellular and systemic levels negatively impacts cell physiology and overall host health. At the cellular level, such deficiency leads to impairment in oxidative energy metabolism, ATP production, disruption in mitochondrial function (mitochondria utilize ∼90% of cellular TPP), oxidative stress, and apoptosis (1, 2, 3, 4, 5). At the systemic level, the deficiency leads to a variety of clinical abnormalities (*e.g.*, neurological and cardiovascular disorders). Vitamin B1 deficiency is not uncommon and occurs in different conditions, including inflammatory bowel disease (IBD) (6), sepsis (7), chronic alcoholism (8, 9), bariatric surgery (10), and diabetes mellitus (11). Optimizing thiamin body level/homeostasis, on the other hand, has been shown to be beneficial in treating disorders like sepsis/septic shock (7, 12) and thiamin-responsive megaloblastic anemia (13); it also reduces fatigue in IBD patients (14).

Humans and other mammals obtain vitamin B1 (*via* intestinal absorption) from exogenous sources as they cannot synthesize it endogenously. Two sources of thiamin are available to the gut: diet and the gut microbiota (15, 16, 17, 18, 19, 20). In the diet, vitamin B1 exists in both the free and phosphorylated forms; the latter form is enzymatically converted (by the action of small intestinal phosphatases) to the free form (*i.e.*, free thiamin) prior to absorption (reviewed in Ref. (20)). Free thiamin is then absorbed by a specific carrier-mediated mechanism that involves two transporters: thiamin transporter-1 (THTR-1) and thiamin transporter-2 (THTR-2); products of the *SLC19A2* and *SLC19A3* genes, respectively; reviewed in Ref. (21)). Microbiota-generated vitamin B1 provides both free thiamin and TPP forms (15, 16, 17), and several phyla (including Bacteroidetes [*Bacteroides fragilis* and *Prevotella copri*], Firmicutes [*Clostridium difficile*, *Ruminococcus lactaris*, and some *Lactobacillus* sp.], Actinobacteria [*Bifidobacterium* sp.], and Fusobacter [*Fusobacterium varium*]) appear to be the main producers of the vitamin (22). Studies with human subjects and rodents have shown that the large intestine is capable of absorbing luminal vitamin B1 (reviewed in Ref. (17); also Refs. (18, 23, 24)). This was confirmed in studies in our laboratory utilizing human colonic epithelial cells and colonic apical membrane vesicle preparations isolated from the colon of organ donors (19, 25, 26). The latter studies have shown that colonic uptake of free thiamin is similar to that in the small intestine and occurs *via* a carrier-mediated process that involves THTR-1 and THTR-2 (19). Colonic uptake of TPP, on the other hand, was found to occur *via* a distinct and an efficient carrier-mediated process that involves the colonic TPP transporter (cTPPT; product of the *SLC44A4* gene (25, 26)). Expression of the cTPPT in the intestinal tract is limited to the large bowel (27, 28) and occurs exclusively at the apical membrane domain of the lining polarized epithelia (25, 26, 27). Since colonocytes have limited ability to generate TPP intracellularly (they express a very low level of the required enzyme, that is, TPKase; (28)), the cTPPT system appears to be of dual importance: it provides the metabolically active colonocytes with their need of an already synthesized TPP and also contributes toward total body nutrition/homeostasis of vitamin B1 (29, 30).

Epithelial cells lining the intestinal tract are exposed to changing levels of oxygen because of their juxtaposition to the oxygen-depleted (anerobic/anoxic) gut lumen and because of the constantly changing rates of oxygen demand/supply (31, 32). This austere microenvironment (considered to be among the most severe in mammalian tissues) leads to a state of hypoxia even under physiologic conditions. Inflammation in the colonic and small intestinal mucosa (as in IBD), however, leads to the development of a more profound and severe form of hypoxia, the so-called pathophysiologic hypoxia (31, 32, 33, 34). Pathophysiologic hypoxia exerts profound effects on the cell physiology including their transport functions; the effect on the latter, however, is differential, and both stimulation and inhibition have been reported (35, 36, 37, 38, 39, 40). Nothing is currently known about the effect of hypoxia on colonic uptake of microbiota-generated vitamin B1 forms (*i.e.*, TPP and free thiamine). We addressed this issue using an *in vitro* (human colonic epithelial NCM460 cells) and an *ex vivo* (human differentiated colonoid monolayers) model. Our results showed that hypoxia causes significant inhibition in colonic uptake of TPP and free thiamin. This inhibition is associated with a marked reduction in level of expression of cTPPT, THTR-1, and THTR-2 and appears to be mediated (at least in part) *via* transcriptional mechanism(s) involving the *SLC44A4*, *SLC19A2*, and *SLC19A3* genes.

## Results

### Effect of hypoxia on colonic uptake of TPP and free thiamin

#### Effect of chamber-induced hypoxia

**Figure 1:**
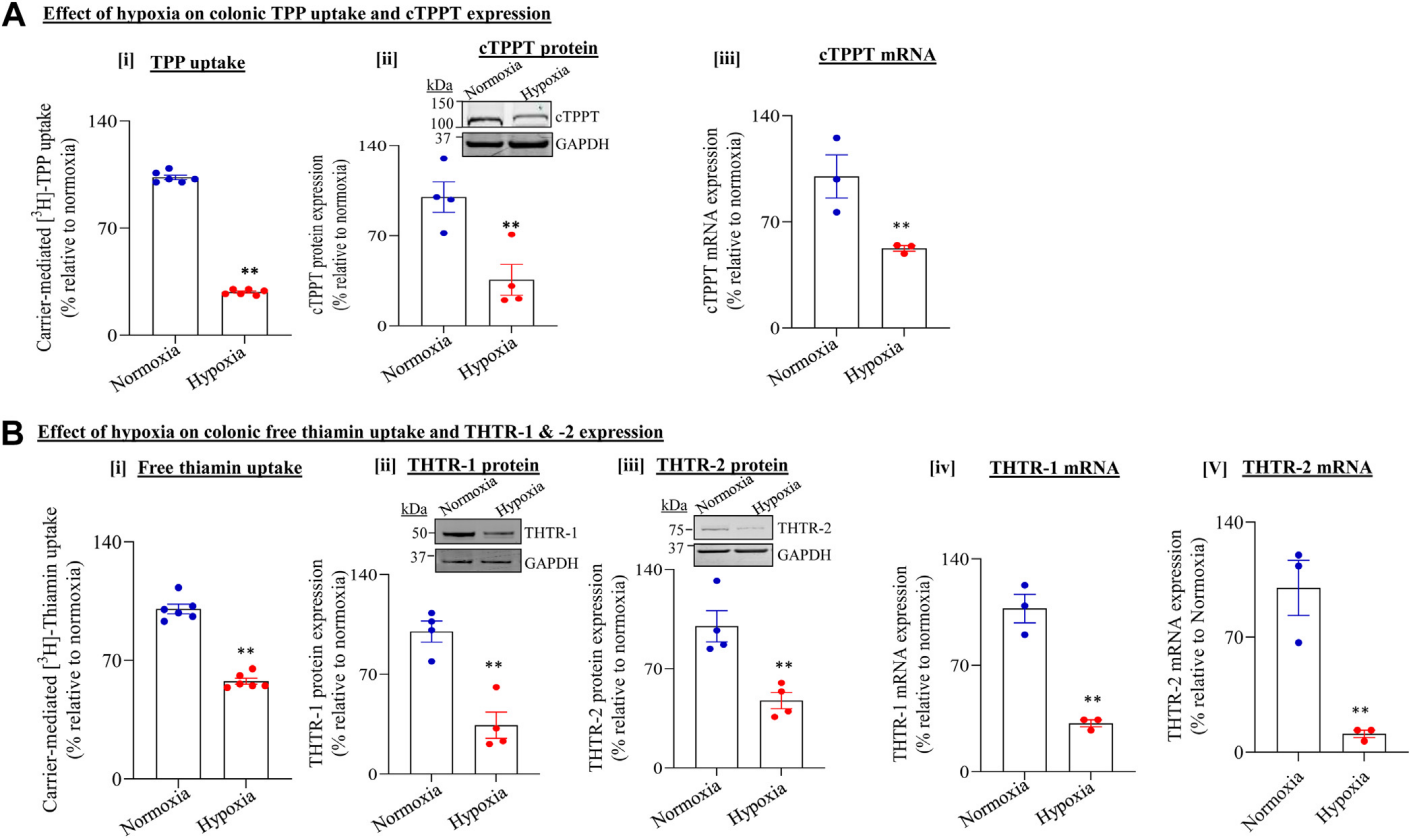
**Effect of chamber-induced hypoxia on TPP and free thiamin uptake by human colonic epithelial NCM460 cells.** Cells were exposed to chamber-induced hypoxia for 16 h. *A*, effect of hypoxia on [i] carrier-mediated [3H]-TPP uptake; [ii] and [iii] level of expression of cTPPT protein and mRNA, respectively. *B*, effect of hypoxia on [i] carrier-mediated [3H]-thiamin uptake; [ii] and [iii] level of expression of THTR-1 and THTR-2 proteins (immunoblot); and [iv] and [v] level of expression of THTR-1 and THTR-2 mRNAs (RT–qPCR), respectively. All protein and mRNA results were normalized relative to GAPDH, and comparison was made relative to simultaneously performed controls (normoxia). Statistical significance of uptake (n = 6; ∗∗*p* < 0.01), protein (n = 4; ∗∗*p* < 0.01), and mRNA (n = 3; ∗∗*p* < 0.01) was evaluated by the Student's *t* test. cTPPT, colonic TPP transporter; THTR-1, thiamin transporter-1; THTR-2, thiamin transporter-2; TPP, thiamin pyrophosphate; qPCR, quantitative PCR.

In these studies, we used *in vitro* and *ex vivo* models to examine the effect of chamber-induced hypoxia on colonic carrier–mediated uptake of TPP and free thiamin. In the *in vitro* model, we exposed human colonic epithelial NCM460 cells to hypoxia by maintaining the cells for 16 h in a hypoxic chamber (see the “Experimental procedures” section). First, we verified that hypoxia was induced in the cells incubated under the hypoxic condition by assessing the level of expression of a positive internal control: phosphoglycerate kinase 1 (PGK-1), an enzyme is known to be induced under hypoxic conditions (41). The results showed a marked induction in level of mRNA expression of marker genes in cells incubated under hypoxic compared with normoxic conditions (mRNA levels of PGK-1 were 100 ± 20 and 370 ± 10 in cells maintained under normoxic and hypoxic conditions, respectively. *p* < 0.05). We then examined the effect of chamber-induced hypoxia on initial rate of carrier-mediated uptake of TPP (0.23 μM) and free thiamin (15 nM) in NCM460. Hypoxia resulted in significant (*p* < 0.01 for both) inhibition in cellular uptake of both TPP and free thiamin relative to normoxia (Fig. 1, *A*-[i] and *B*-[i]). In both cases, the inhibition was found to be associated with a marked reduction in level of expression of the involved transporters (*i.e.*, cTPPT for TPP and THTR-1 and THTR-2 for free thiamin) at both the protein and mRNA levels (Fig. 1, *A*-[ii] and [iii] and *B*-[ii]–[v]).

**Figure 2:**
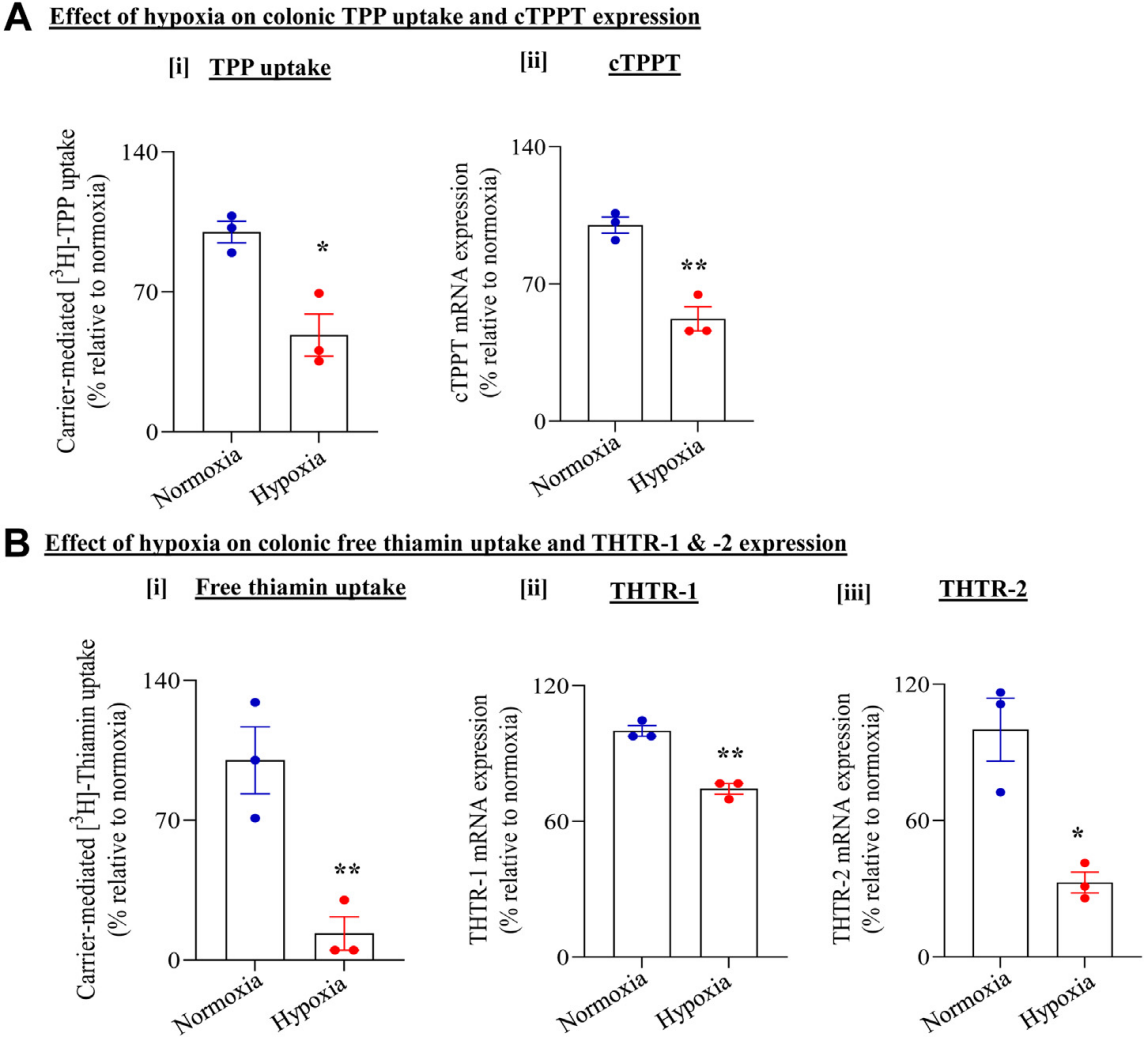
**Effect of chamber-induced hypoxia on TPP and free thiamin uptake by human differentiated colonoid monolayers.** Colonic monolayers were exposed to chamber-induced hypoxia for 16 h. *A*, effect of hypoxia on [i] colonic carrier–mediated [^3^H]-TPP uptake; [ii] level of expression of cTPPT mRNA. *B*, effect of hypoxia on [i] colonic carrier–mediated [^3^H]-thiamin uptake; [ii] and [iii] level of expression of THTR-1 and THTR-2 mRNAs, respectively. All mRNA results were normalized relative to GAPDH, and comparison was made relative to simultaneously performed controls (normoxia). Statistical significance of all uptake and mRNA data (n = 3 for all; ∗∗*p* < 0.01; ∗*p* < 0.05, respectively) was evaluated by the Student's *t* test. cTPPT, colonic TPP transporter; THTR-1, thiamin transporter-1; THTR-2, thiamin transporter-2; TPP, thiamin pyrophosphate.

To establish translational relevance to the findings on the effect of hypoxia on colonic carrier–mediated TPP and free thiamin uptake observed with cultured NCM460 cells, we examined the effect of chamber-induced hypoxia on carrier-mediated uptake of these two forms of vitamin B1 using the *ex vivo* human differentiated colonoid monolayers. The results again showed significant inhibition in carrier-mediated TPP (*p* < 0.05) and free thiamin (*p* < 0.01) uptake by colonoid monolayers maintained under hypoxic compared with normoxic conditions. This inhibition was similarly associated with a significant reduction in mRNA expression of the respective transporters (for cTPPT, *p* < 0.01; THTR-1, *p* < 0.01; and THTR-2, *p* < 0.05) in hypoxia-exposed colonoid monolayers compared with normoxia (Fig. 2, *A* and *B*).

Taken together, the aforedescribed findings show that exposure of human colonic epithelial cells to hypoxia significantly impairs uptake of the microbiota-generated TPP and free thiamin and that the effect is mediated *via* reduction in level of expression of their respective uptake systems.

#### Effect of chemically induced hypoxia

**Figure 3:**
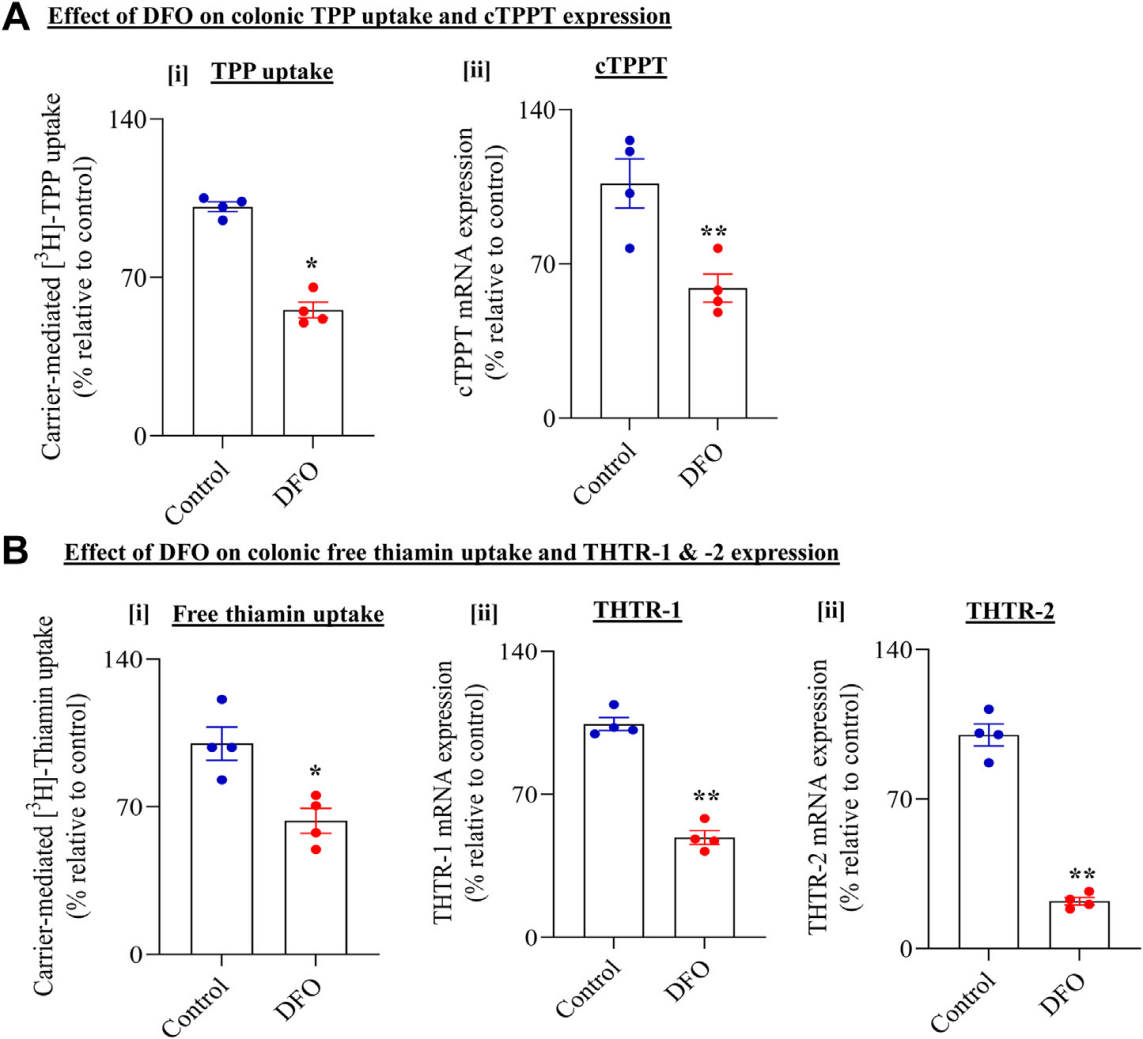
**Effect of chemically induced hypoxia on uptake of TPP and free thiamin by human colonic epithelial NCM460 cells.** Cells were treated with the hypoxia-mimetic agent DFO (250 μM) for 48 h. *A*, effect of hypoxia on [i] colonic carrier–mediated [^3^H]-TPP uptake and [ii] level of expression of cTPPT mRNA. *B*, effect of hypoxia on [i] colonic carrier–mediated [^3^H]-thiamin uptake; [ii] and [iii] level of expression of THTR-1 and THTR-2 mRNA, respectively. All mRNA results were normalized relative to GAPDH, and comparison was made relative to simultaneously performed controls (normoxia). Statistical significance of uptake (n = 4; ∗*p* < 0.05) and mRNA (n = 4; ∗∗*p* < 0.01) was evaluated by the Student's *t* test. cTPPT, colonic TPP transporter; DFO, desferrioxamine; THTR-1, thiamin transporter-1; THTR-2, thiamin transporter-2; TPP, thiamin pyrophosphate.

In these studies, we examined the effect of chemically induced hypoxia on initial rate of colonic carrier–mediated uptake of TPP and free thiamin. For this, we treated human colonic epithelial NCM460 cells with the hypoxia-mimetic agent desferrioxamine (DFO) (250 μM for 48 h; (38)) followed by transport investigations. First, we verified induction of hypoxia in the DFO-treated cells by assessing level of expression of two positive internal controls: lactate dehydrogenase A (LDHA) and PGK-1 (41). DFO treatment resulted in a marked induction in mRNA expression of both enzymes compared with untreated control cells. (Relative expression levels of LDHA: 100 ± 5 and 598 ± 33 and PGK-1: 100 ± 2.4 and 650 ± 18 in control and DFO-treated cells, respectively; *p* < 0.01 for both). We then examined the effect of chemically induced hypoxia on carrier-mediated TPP (0.23 μM, 7 min) and free thiamin (15 nM, 10 min) uptake. Again, we observed significant (*p* < 0.05 for both) inhibition in carrier-mediated TPP and free thiamin uptake in DFO-treated cells compared with untreated controls (Fig. 3, *A*-[i] and *B*-[i]). This inhibition was associated with marked (*p* < 0.01 for all) reduction in level of mRNA expression of cTPPT, THTR-1, and THTR-2 in hypoxic compared with normoxic cells (Fig. 3, *A*-[ii] and *B*-[ii]–[iii]).

Collectively, the aforementioned findings show that chamber-induced and chemically induced hypoxia inhibits colonic carrier–mediated uptake of TPP and free thiamin, and that the inhibition is associated with reduction in level of expression of TPP and free thiamin transporters.

### Effect of hypoxia on the activity of the *SLC44A4*, *SLC19A2*, and *SLC19A3* gene promoters in colonic epithelial cells: involvement of transcriptional mechanism(s)

**Figure 4:**
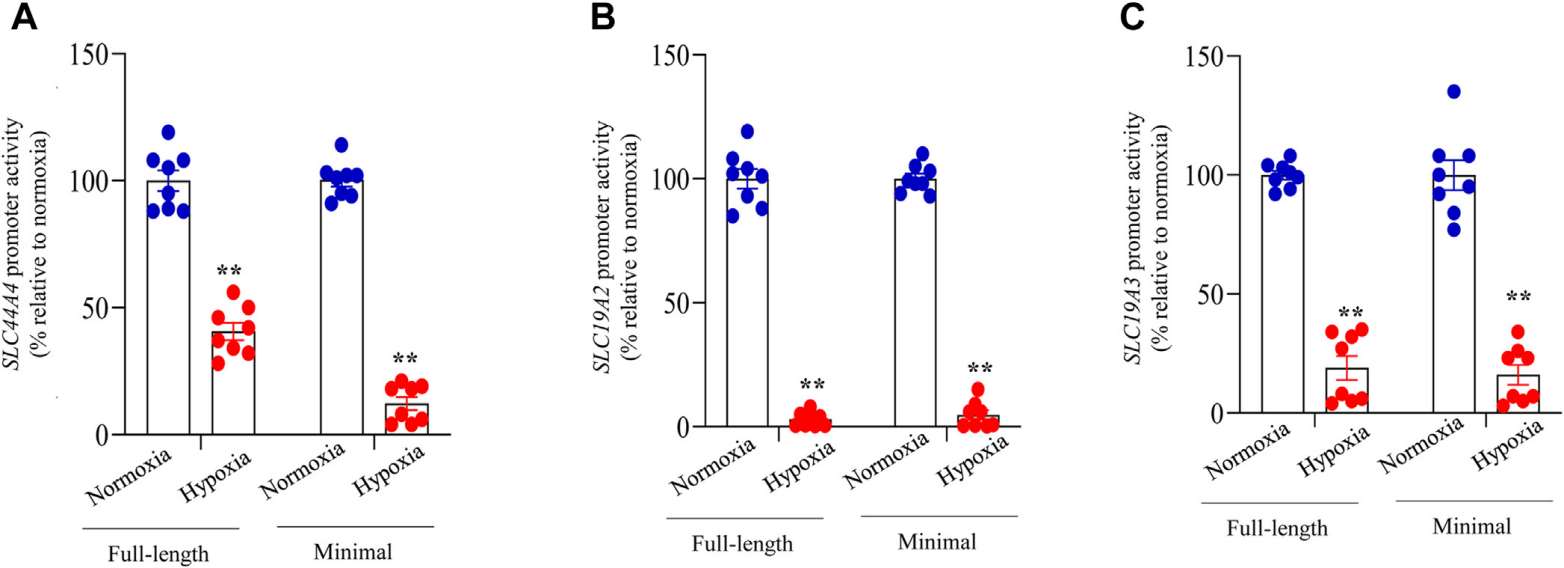
**Effect of exposure of human colonic epithelial NCM460 cells to chamber-induced hypoxia on promoter activity.***A*, *SLC44A4*; *B*, *SLC19A2*; and *C*, *SLC19A3* genes. Full-length and minimal-promoter constructs of the respective genes in pGL3 basic vector with pRLTK plasmid were transfected into NCM460 cells. Forty-eight hours following transfection, cells were exposed to chamber-induced hypoxia for additional 16 h followed by the determination of luciferase activity. All luciferase activities were normalized relative to PGL3 basic vector, and comparison was made relative to simultaneously performed controls (normoxia). Statistical significance of all data (n = 8 for all; ∗∗*p* < 0.01) were evaluated by the Student’s *t* test. pRLTK, thymidine kinase promoter-Renilla luciferase.

The aforeobserved inhibition in colonic carrier–mediated uptake of TPP and free thiamin and in the level of mRNA expression of the involved transporters suggests possible involvement of transcriptional mechanism(s) involving the *SLC44A4*, *SLC19A2*, and *SLC19A3* genes. To test this possibility, we exposed colonic epithelial NCM460 cells transfected with *SLC44A4*, *SLC19A2*, and *SLC19A3* full-length (as well as minimal) promoters fused to the luciferase reporter gene to chamber-induced hypoxia followed by examination of promoter activities. Exposure of cells to hypoxia resulted in significantly (*p* < 0.01 for all) reduced activity of *SLC44A4*, *SLC19A2*, and *SLC19A3* full-length (as well as minimal) promoters compared with normoxic conditions (Fig. 4, *A*–*C*). These findings suggest that transcriptional mechanisms mediate the effects of hypoxia on carrier-mediated TPP and free thiamin uptake by human colonocytes. The data also demonstrate that the hypoxia-responsive regions are in the minimal promoter regions of these genes.

### Effect of chamber-induced hypoxia on level of expression of hypoxia-inducible transcription factors in human colonocytes

**Figure 5:**
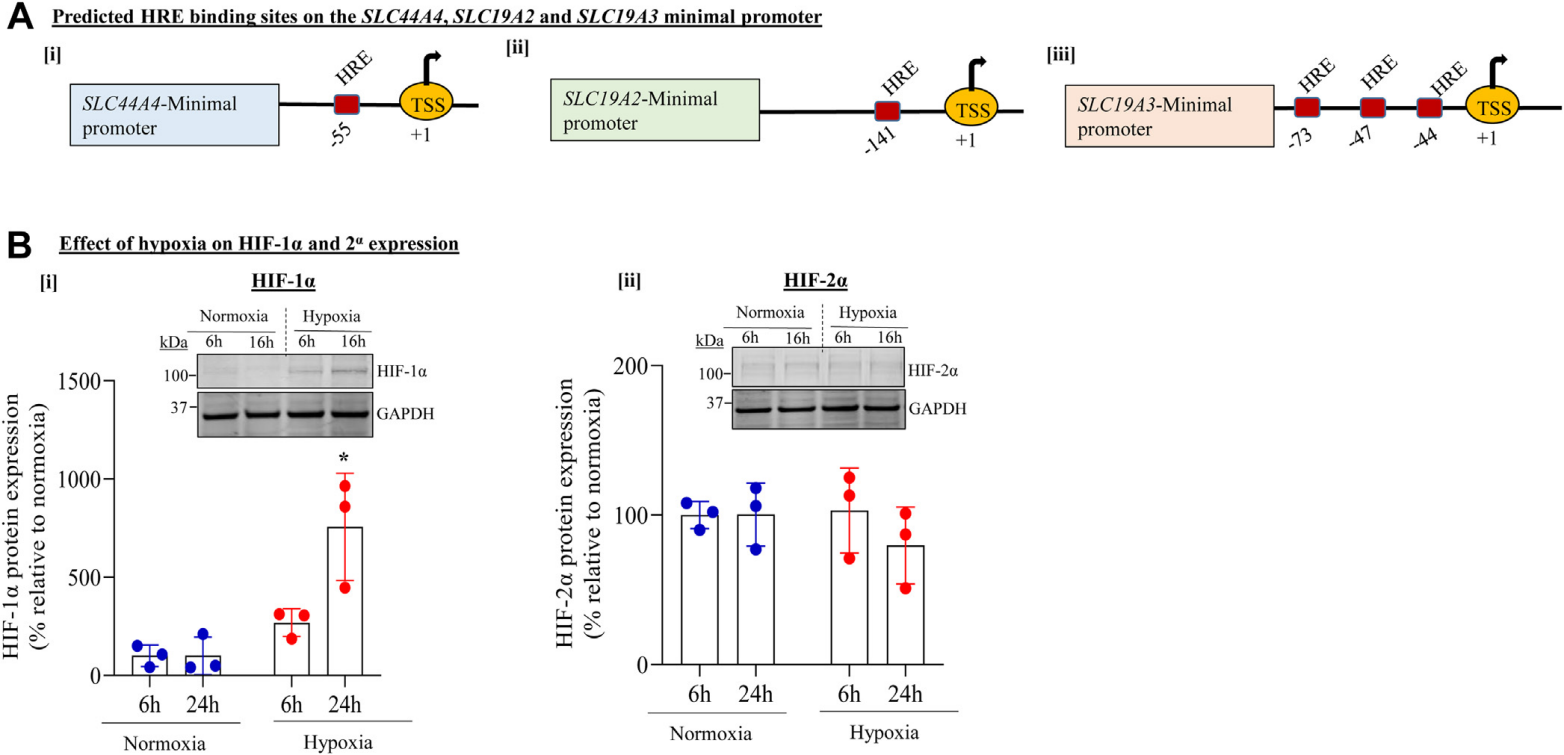
**Effect of hypoxia on chamber-induced hypoxia on level of expression of hypoxia-inducible factors (HIFs) in human colonic epithelial NCM460 cells.***A*, schematic representation of the [i] *SLC44A4*, [ii] *SLC19A2*, and [iii] *SLC19A3* minimal promoter regions depicting predicted binding sites for HREs. *B*, NCM460 cells were exposed to chamber-induced hypoxia for 16 h followed by examination of HIF expression. Level of expression of HIF-1α and HIF-2α protein (immunoblot) in hypoxia-exposed cells compared with those exposed to normoxia (control) condition. All protein results were normalized relative to GAPDH. Statistical significance of all proteins (n = 3; ∗*p* < 0.05) was evaluated using the Student's *t* test. HRE, hypoxia-responsive element.

Cellular responses to hypoxia are primarily mediated by hypoxia-inducible transcription factors (HIFs), especially HIF-1α and HIF-2α (42, 43). In this study, we examined the effect of chamber-induced hypoxia on level of expression of these factors in human colonic epithelial NCM460 cells. First, we subjected the *SLC44A4*, *SLC19A2*, and *SLC19A3* minimal promoter regions to computer analysis (Eukaryotic Promoter Database; https://epd.epfl.ch//index.php) to determine if they contain putative hypoxia-responsive element (HRE)–binding sites (*i.e.*, “R-CGTG”). The computational analysis predicted the *SLC44A4* and *SLC19A2* minimal promoters to contain one putative HRE sequence at position −55 and −141, respectively. The minimum promoter of *SLC19A3*, on the other hand, was found to contain three such putative HRE sequences located at positions −44, −47, and −73 (Fig. 5*A*). We then examined (by immunoblotting) the effect of chamber-induced hypoxia on level of expression of HIF-1α and HIF-2α proteins in human colonic epithelial NCM460 cells; results were compared with their level of expression in control cells maintained under normoxic condition. Detectable levels of HIF-1α in hypoxic cells (but not those maintained under normoxic condition) increased with time with hypoxia (Fig. 5*B*-[i]). On the other hand, the very low level of HIF-2α protein detected in colonic NCM460 cells maintained under hypoxic conditions was similar to that in cells maintained under control (normoxic) conditions (Fig. 5*B*-[ii]). These findings suggest a role for HIF-1α in mediating the inhibitory effect of hypoxia on colonic uptake of TPP and free thiamin.

### Role of HIF-1α in mediating the inhibitory effect of hypoxia on colonic uptake of TPP and free thiamin and on expression of cTPPT, THTR-1, and THTR-2

**Figure 6:**
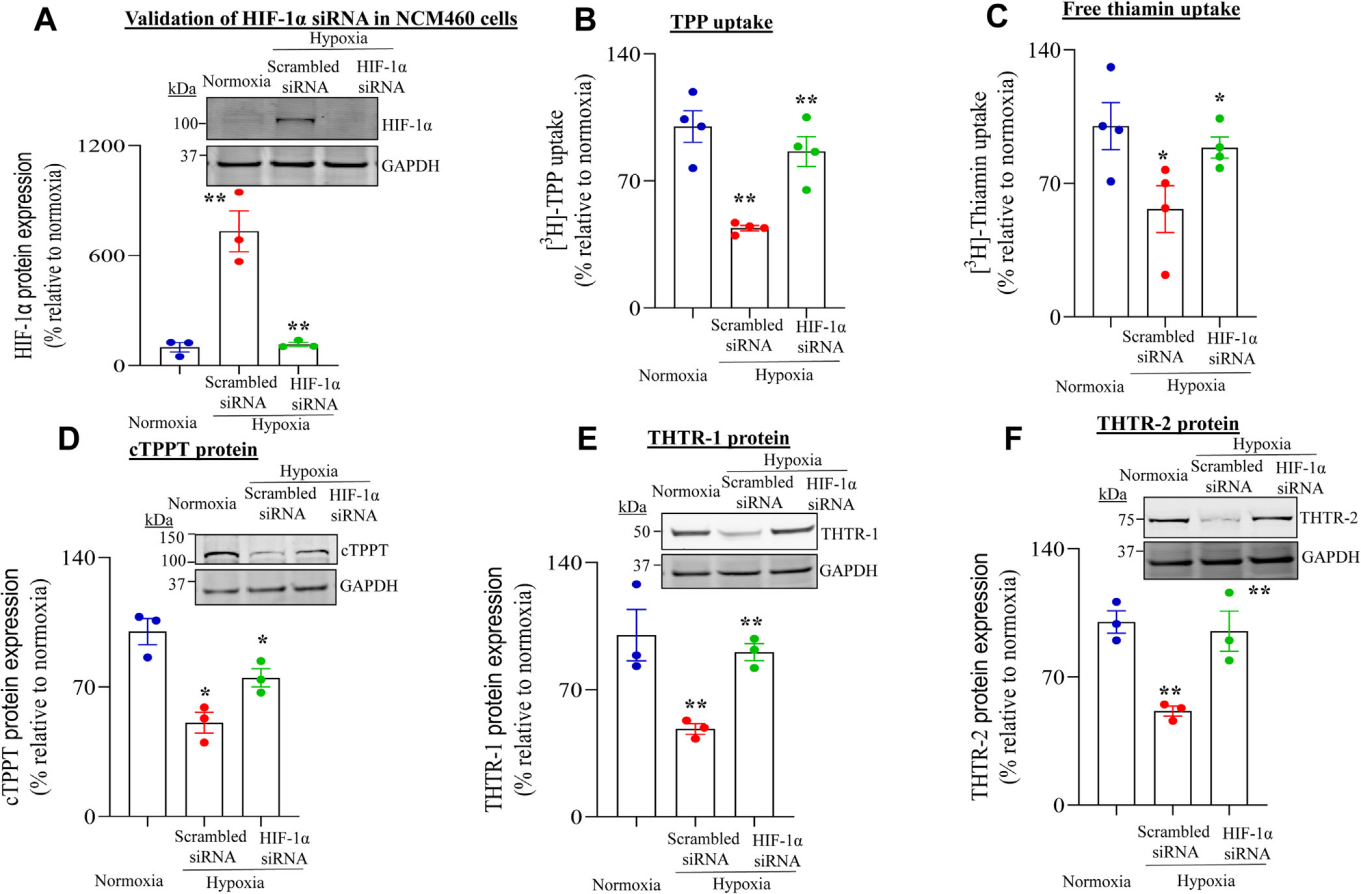
**Effect of hypoxia on knocking down HIF-1α with gene-specific siRNA on the inhibitory effect of hypoxia on uptake of TPP and free thiamin and on the level of expression of cTPPT, THTR-1, and THTR-2 in human colonic epithelial NCM460 cells.** Cells were transfected with prevalidated HIF-1α-specific or scrambled (nontargeting negative control) siRNAs (42) as described in the Experimental procedures section. Twenty four hours following transfection, cells were exposed to normoxic or hypoxic conditions for an additional 16 h. *A*, immunoblot showing the effect of transfection with HIF-1α-specific and scrambled siRNAs on level of expression of the HIF-1α protein (probing was done using specific anti-HIF-1α antibody). *B*, initial rate (7 min) of carrier-mediated [^3^H]-TPP uptake. *C*, initial rate (10 min) of carrier-mediated [^3^H]-thiamin uptake. *D*–*F*, level of expression of cTPPT, THTR-1, and THTR-2 proteins (immunoblot) probed with the transporter-specific antibodies (see the Experimental procedures section). All protein results were normalized relative to GAPDH, and comparison was made relative to simultaneously performed controls (normoxia). Statistical significance of uptake (n = 4; ∗∗*p* < 0.01; ∗*p* < 0.05) and protein (n = 3; ∗∗*p* < 0.01; ∗*p* < 0.05) data was evaluated by the Student's *t* test. cTPPT, colonic TPP transporter; HIF-1α, hypoxia-inducible transcription factor 1α; THTR-1, thiamin transporter-1; THTR-2, thiamin transporter-2; TPP, thiamin pyrophosphate.

To further confirm the contribution of HIF-1α in mediating the inhibitory effect of hypoxia on carrier-mediated colonic TPP and free thiamin uptake and expression of their relevant transporters, we examined the effect of siRNA silencing on the *HIF-1α* gene (44) in NCM460 cells maintained under chamber-induced hypoxia on their ability to take up TPP and free thiamin. First, we established that in hypoxic NCM460 cells, HIF-1α gene-specific siRNA leads to significant (*p* < 0.01) inhibition in level of expression of HIF-1α protein (Fig. 6*A*). Under chamber-induced hypoxia, HIF-1α-specific siRNA treatment of NCM460 cells led to a significant (*p* < 0.01–0.05) recovery in transport of both TPP and free thiamin substrates relative to uptake by cells treated with scrambled siRNAs (Fig. 6, *B* and *C*). Similarly, expression of the corresponding cTPPT (*p* < 0.05), THTR-1 (*p* < 0.01), and THTR-2 (*p* < 0.01) proteins was enhanced under hypoxic conditions in NCM460 cells pretreated with HIF-1α siRNAs compared with cells pretreated with scrambled siRNA (Fig. 6, *D*–*F*). These findings demonstrate that HIF-1α mediates the inhibitory effects of hypoxia on colonic carrier–mediated uptake of TPP and free thiamin and on levels of expression of their relevant transporters.

### Effect of hypoxia on expression of nuclear factors required for basal activity of the *SLC44A4*, *SLC19A2*, and *SLC19A3* gene promoters

**Figure 7:**
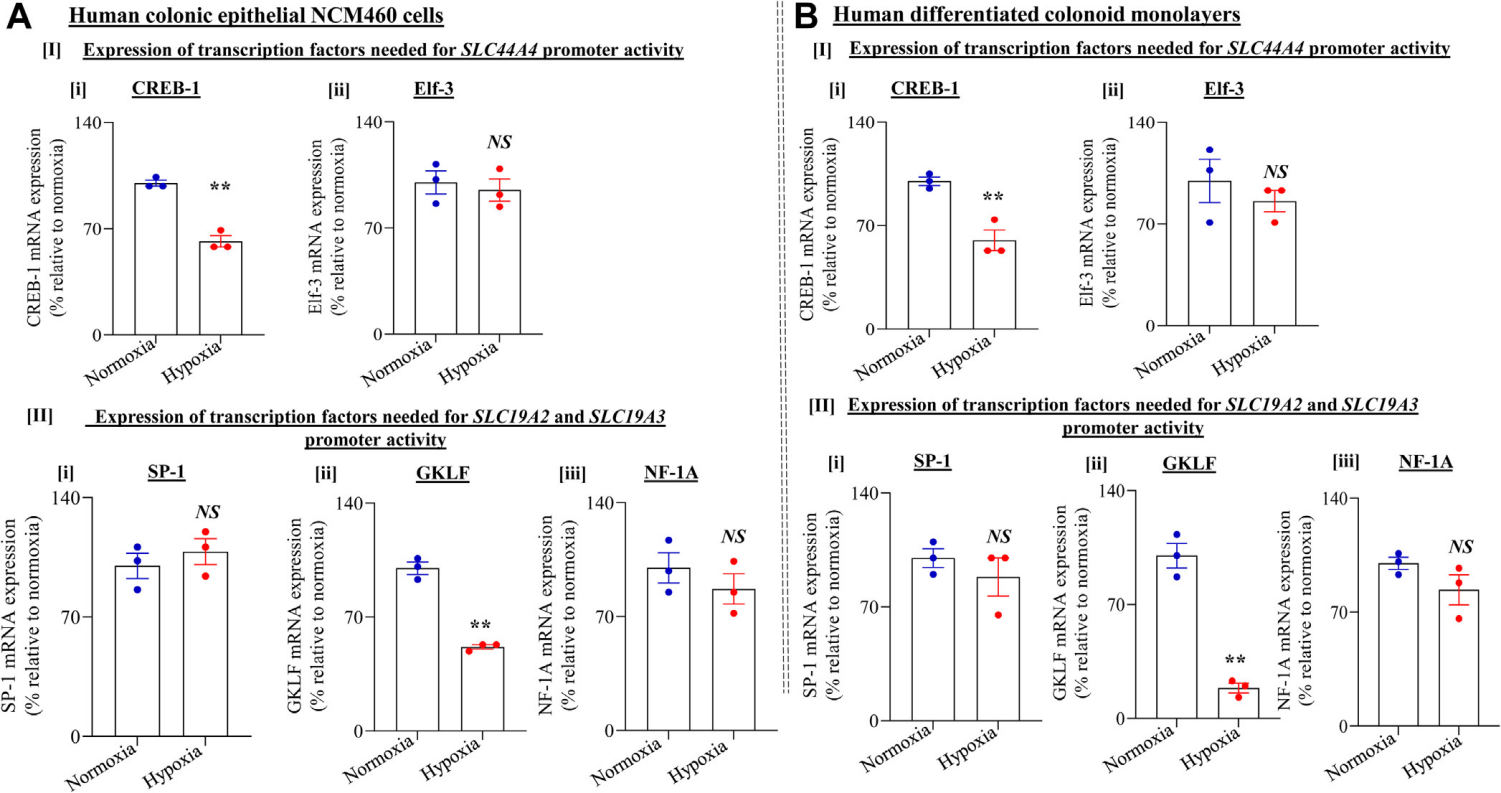
**Effect of chemically induced hypoxia on expression of transcription factors (TFs) that regulate activity of the *SLC44A4*, *SLC19A2*, and *SLC19A3* gene promoters.***A*, human colonic epithelial NCM460 cells. *B*, human differentiated colonoid monolayers. NCM460 cells and human differentiated colonoid monolayers were exposed to hypoxic (or normoxic) conditions, followed by determination of levels of mRNA expression of the TFs CREB-1 and Elf-3 (for *SLC44A4* promoter) as well as SP1, GKLF4, and NF-1A (for *SLC19A2* and *SLC19A3* promoters). All mRNA expression data were normalized relative to GAPDH and compared with their respective controls (n = 3; ∗∗*p* < 0.01). Statistical significance was evaluated by Student’s *t* test. CREB-1, cAMP responsive element–binding protein 1; Elf-3, E74-like ETS transcription factor 3; GKLF-4, gut-enriched Kruppel-like factor 4; NF-1A, neurofibromatosis-1A; NS, not significant; SP1, specificity protein 1.

The aforedescribed studies demonstrate that hypoxia negatively impacts colonic uptake of TPP and free thiamin as well as expression of their transporters, and that the effect is mediated *via* transcriptional modulation of the *SLC44A4*, *SLC19A2*, and *SLC19A3* genes. Whether part of the effect of hypoxia on transcription of these genes is also mediated through inhibited expression of nuclear factor(s) required for basal activity of their promoters is not clear. Thus, we examined the effect of chamber-induced hypoxia on level of expression of the transcription factors cAMP responsive element–binding protein (CREB), E74-like ETS transcription factor 3 (Elf-3), specificity protein 1 (SP-1), gut-enriched Krüppel-like factor 4 (GKLF-4), and neurofibromatosis-1 (NF-1) in human colonic NCM460 cells as well as human differentiated colonoid monolayers. We focused on these transcription activators because previous studies from our laboratory have shown that the minimal promoter regions of the *SLC44A4*, *SLC19A2*, and *SLC19A3* genes contain *cis*-elements for these nuclear factors that are important for their activity (the minimal promoter region of *SLC44A4* contains CREB [−35] and Elf-3 [−77]; that of *SLC19A2* contains SP-1 [−238], GKLF-4 [−305], NF-1 [−350]; and that of *SLC19A3* contains an SP-1 [−45]) (43, 44, 45). Results of the quantitative PCR (qPCR) analysis showed significant (*p* < 0.01 for both) reduction in level of mRNA expression of the CREB-1 and GKLF-4 (but not that of Elf-3, SP-1, or NF-1A) in human colonic epithelial NCM460 cells (Fig. 7*A*) as well as human colonoid monolayers (Fig. 7*B*) maintained under hypoxic condition compared with those maintained under normoxic condition.

## Discussion

Our aims in these investigations were to examine the effects of pathophysiological hypoxia on colonic uptake of the microbiota-generated TPP as well as free thiamin. As mentioned earlier, hypoxia exerts profound effects on cell physiology including transport events at their cell membranes (35, 36, 37, 38, 39, 40). Nothing, however, is known about the effect of hypoxia on colonic uptake of vitamin B1, a micronutrient that is essential for cellular energy metabolism/ATP production, mitochondrial function, and for reduction of oxidative stress (1, 2, 3, 4). We investigate this issue utilizing two complementary models of human colonic epithelial cells: an *in vitro* (human epithelial NCM460 cells) and an *ex vivo* (human differentiated colonoid monolayers) model. Also, we employed two models of hypoxia in our investigations: chamber-induced as well as chemically induced hypoxia.

Results of our investigations showed that exposure of human colonic epithelial NCM460 cells to chamber-induced or to chemically induced hypoxia lead to a significant inhibition in carrier-mediated TPP and free thiamin uptake. This inhibition was associated with a significant reduction in level of expression of cTPPT as well as THTR-1 and THTR-2 proteins and mRNAs. Similar findings were observed when human differentiated colonoid monolayers were exposed to chamber-induced hypoxia, where a significant inhibition in carrier-mediated TPP and free thiamin uptake occurred that was associated with a significant reduction in level of expression of cTPPT and THTR-1 and THTR-2.

The inhibitory effects of hypoxia on colonic TPP and free thiamin uptake and expression of their transporters were found to be mediated (at least in part) *via* transcriptional mechanisms affecting the respective genes of the involved transporters. This was evident from the results of studies of the effect of chamber-induced hypoxia on activity of the *SLC44A4*, *SLC19A2*, and *SLC19A3* promoters (both full-length and minimal promoters), where significant inhibition in activity of these promoters was observed in colonic epithelial cells exposed to hypoxia compared with those maintained under normoxic conditions. The fact that hypoxia caused a similar degree of inhibition in activity of full-length as well as minimal promoters of these genes suggests that the HREs are located in latter regions. Indeed, interrogation of the minimal promoter regions of *SLC44A4*, *SLC19A2*, and *SLC19A3* by the Eukaryotic Promoter Database program predicted existence of HREs in all these regions. Since cellular responses to hypoxia are mainly mediated by HIF-1α and HIF-2α (42, 43), we examined the effect of chamber-induced hypoxia on level of expression of these factors in human colonic epithelial NCM460 cells. The results showed induction in level of expression of HIF-1α (but not HIF-2α) in cells exposed to hypoxia, which increased with time. These findings suggested a role for HIF-1α in mediating the effect of hypoxia on colonic TPP and free thiamin uptake and expression of their respective transporters. The latter suggestion was confirmed in the study examining the effect of knocking down HIF-1α of NCM460 cells with gene-specific siRNAs on the hypoxia-mediated inhibition of TPP and free thiamin uptake and expression of their transporters. Indeed, a significant reversal in the inhibitory effect of hypoxia on colonic TPP and free thiamin uptake as well as on level of expression of cTPPT, THTR-1, and THTR-2 proteins was observed as a result of HIF-1α knockdown. These findings clearly establish a role for HIF-1α in mediating the inhibitory effect of hypoxia on colonic uptake of TPP and free thiamin.

Previous studies from our laboratory have established that transcription factors, CREB-1, Elf-3, GKLF4, NF-1, and SP1, play an important role in driving the activity of the *SLC44A4*, *SLC19A2*, and *SLC19A3* promoters (45, 46, 47). Thus, we also examined whether part of the hypoxia effects on colonic TPP and free thiamin uptake and expression of their transporters could also involve suppression in level of expression of these nuclear factors. The results indeed showed significant suppression in level of expression of CREB-1 and GKLF-4 (with no effect on level of expression of Elf-3, SP-1, or NF-1A) in NCM460 cells and human differentiated colonoid monolayers exposed to hypoxia compared with those maintained under normoxic condition. These findings suggest possible involvement of CREB-1 and GKLF4 in mediating (part of) the inhibitory effect of hypoxia on cTPPT and THTR-1 expression and ultimately colonic TPP and thiamin uptake. Whether the reduction in level of expression of the latter transcription factors is mediated *via* HIF-1α or other hypoxia-mediated mechanisms is not known at this stage requires further investigations.

Our findings of inhibition in uptake of free thiamin and expression of its transporters in human colonocytes are in contrast to the stimulatory effect of hypoxia on vitamin B1 uptake and expression of THTR-1 observed in breast cancer cells (38). This suggests that the effects of hypoxia on vitamin B1 transport are cell specific in nature. In summary, our findings show for the first time that exposure of human colonocytes to hypoxia negatively impacts the transport physiology and molecular biology of TPP and free thiamin uptake processes. The results also show that these effects are mediated (at least in part) at the level of transcription of the *SLC44A4*, *SLC19A2*, and *SLC19A3* genes.

## Experimental procedures

### Chemicals and reagents

[^3^H]-TPP (specific activity: >1.4 Ci/mmol; radiochemical purity: >98.2%) and [^3^H]-thiamin (specific activity: >12.8 Ci/mmol; radiochemical purity: >93.3%) were purchased from Moravek, Inc. Human-specific anti-TPPT (*SLC44A4*) affinity-purified rabbit polyclonal antibody was generated for us by Thermo Fisher Scientific; anti-THTR-1 (catalog no.: ab229680) rabbit polyclonal antibody was from Abcam; anti-THTR-2 (catalog no.: 13407-1-AP) rabbit polyclonal antibody was from Proteintech; anti-HIF-1α (catalog no.: 14179S) rabbit monoclonal antibody was from Cell Signaling Technology; anti-HIF-2α (catalog no.: NB100-122) rabbit polyclonal antibody was from Novus Biologicals; and anti-GAPDH mouse monoclonal antibody (catalog no.: sc-47724) was from Santa Cruz. The secondary antibodies, anti-rabbit IRDye-800 (catalog no.: 926-32211) and antimouse IRDye-680 (catalog no.: 926-68020), were purchased from LI-COR Bioscience. All other chemicals and reagents used in this studies were of analytical/molecular biology grade and purchased from established sources.

### Culturing of human-derived colonic epithelial NCM460 cells and exposure to hypoxia

NCM460 cells were obtained from INCELL and maintained in M3 base culture medium, supplemented with fetal bovine serum (20%), penicillin (100 U/ml), and streptomycin (100 μg/ml) and incubated at 37 °C in 5% CO_2_ incubator as described before. Our selection of the NCM460 cells as the model in the current investigation is based on the fact that they are similar to native human colonic preparations in that they transport TPP and free thiamin *via* distinct and specific carrier-mediated processes (19, 21, 25, 26). Also, our findings on the effect of hypoxia were not unique to these cells but were also observed with the human colonic epithelial CCD 841 cells (data not shown). In these studies, we grew the NCM460 cells to 70 to 80% confluency, then maintained them (for the indicated periods) under either a standard normoxic, that is, control (humidified air with 5% CO_2_; ThermoFisher Forma Series II incubator) or hypoxic (1% O_2_, 94% N_2_, and 5% CO_2_; Thermo Scientific HERACELL150i incubator) conditions. Chemical hypoxia was induced in NCM460 cells by incubating them in the presence of 250 μM DFO (a hypoxic-memetic agent that chelates iron and prevents the degradation of HIF-1α protein leading to hypoxia; (38, 48)) for 48 h; control cells were run simultaneously and maintained in the absence of DFO.

### Culturing of human differentiated colonoid monolayers and exposure to hypoxia

Human colonoids were prepared from biopsy samples of adult healthy individuals at the Digestive Diseases Research Center of the Washington University, School of Medicine and were grown as previously described (49, 50). Briefly, isolated colonoids were thawed and plated (in 24-well plates) in Matrigel (BD Biosciences) droplets (15 μl) followed by incubation at 37 °C with conditioned media (a 1:1 mixture of the L-WRN cell line and primary culture media [Advanced DMEM/F12; Invitrogen] supplemented with fetal bovine serum [20%], l-glutamine [2 mM], penicillin [100 U/ml], streptomycin [100 μg/ml], Stemolecule Y27632 [10 μM; Reprocell], and SB 431542 [10 μM; Peprotech]). To obtain polarized differentiated colonoid monolayers, cells were plated at density of 5 × 10^4^ cells/well onto 24-well cell culture inserts (Corning) coated with type IV human collagen (Sigma) and then grown for 4 days prior to induction of differentiation by addition of differentiation media (5% conditioned media added with only Y-27632 inhibitor). Differentiated colonoid monolayers were then exposed as indicated to normoxia and hypoxia conditions as mentioned previously.

### Carrier-mediated [^3^H]-TPP and [^3^H]-thiamin uptake

Initial rates (10 min for *in vitro*; 30 min for *ex vivo*; both at 37 °C) of carrier-mediated TPP and free thiamin uptake were examined in normoxic and hypoxic NCM460 cells (*in vitro*) as well as differentiated colonoid monolayers (*ex vivo*) incubated in Krebs–Ringer buffer (pH 7.4) containing [^3^H]-TPP (0.23 μM) or [^3^H]-thiamin (15 nM). At the end of incubations, cells/monolayers were then washed with ice-cold Krebs–Ringer buffer followed by lysis with NaOH and neutralization with 10 N HCl. The radioactive content was counted using a liquid scintillation counter as described previously (50). Uptake of TPP and free thiamin by their respective and distinct carrier-mediated mechanism was determined by subtracting uptake of [^3^H]-TPP or free [^3^H]-thiamin in the presence of a high pharmacological concentration (1 mM) of unlabeled TPP or free thiamin from uptake in their absence; all uptake data points were calculated relative to total protein content (in milligrams) of the different preparations.

### siRNA transfection

A well-validated silencer-select siRNA targeting HIF-1α (s6541) and negative control siRNA (catalog no.: 4390843) (44) were obtained from Thermo Fisher Scientific. NCM460 cells were transfected with siRNAs for 24 h using Lipofectamine RNAiMAX transfection reagent (Invitrogen) following the manufacturer's instructions. During transfection, the culture medium containing the siRNA and transfection reagent was changed daily. After transfection, the cells were exposed to normoxic or hypoxic conditions for an additional 16 h.

### Isolation of RNA, complementary DNA synthesis, and real-time qPCR assay

Total RNA was isolated from human colonic epithelial NCM460 cells and human differentiated colonoid monolayers using QIAzol Lysis reagent (QIAGEN) and RNeasy Kit (QIAGEN) as previously described (50). The RNA was then converted to complementary DNA using the Verso-cDNA Synthesis Kit (Thermo Fisher Scientific), and levels of mRNA expression of *SLC44A4*, *SLC19A2*, *SLC19A3*, CREB-1, Elf-3, SP-1, GKLF, NF-1, PGK-1, and LDHA were then quantified by real-time qPCR using iQ SYBER Green Super mix (Bio-Rad) in the CFX96 real-time PCR system (Bio-Rad) following the manufacturer's guidelines using gene-specific primers (Table 1). Relative gene expression was quantified by normalizing threshold cycle (Ct) values to the respective GAPDH following 2^−ΔΔCt^ method (51).

**Table 1:** List of primer sequences for RT–qPCR

Gene name	Forward primers (5′–3′)	Reverse primers (5′—3′)
Human cTPPT	TGCTGATGCTCATCTTCCTGCG	GGACAAAGGTGACCAGTGGGTA
Human THTR-1	GCCAGACCGTCTCCTTGTA	TAGAGAGGGCCCACCACAC
Human THTR-2	TTCCTGGATTTACCCCACTG	GTATGTCCAAACGGGGAAGA
Human CREB-1	TTAACCATGACCAATGCAGCA	TGGTATGTTTGTACGTCTCCAGA
Human Elf-3	TCTTCCCCAGCGATGGTTTTC	TCCCGGATGAACTCCCACA
Human SP-1	CCATACCCCTTAACCCCG	GAATTTTCACTAATGTTTCCCACC
Human GKLF-4	CCGCTCCATTACCAAGAGCT	ATCGTCTTCCCCTCTTTGGC
Human NF-1A	GCAGGCCCGAAAACGAAAATA	TTTGCCAGAAGTCGAGATGCC
Human GAPDH	CGACCACTTTGTCAAGCTCA	AGGGGAGATTCAGTGTGGTG
Human PGK-1	TTAAAGGGAAGCGGGTCGTTA	TCCATTGTCCAAGCAGAATTTGA
Human LDHA	CTCCAAGCTGGTCATTATCACG	AGTTCGGGCTGTATTTTACAACA

### Isolation of protein and immunoblotting assay

Total protein was isolated from human colonic NCM460 cells using radioimmunoprecipitation assay buffer (Sigma) with protease inhibitor cocktail, and an equal amount (45 μg) of the proteins was loaded on a NuPAGE 4 to 12% Bris–Tris gradient minigels (Invitrogen) as previously described (47). The proteins were then blotted onto polyvinylidene difluoride membranes and probed with anti-cTPPT (1:500), anti-THTR-1 (1:1000), anti-THTR-2 (1:1000), anti-HIF-1α (1:500), or anti-HIF-2α (1:500) antibodies and simultaneously with anti-GAPDH (1:2000) primary antibodies. Specificity of the anti-cTPPT, THTR-1, and THTR-2 antibodies was validated in our laboratory previously using different approaches that include overexpression of tag protein or gene silencing (52, 53). Other antibodies were validated by either the respective company or by the other investigators using knockout animals and/or gene knockdown approaches. The immune-reactive bands from the blot were then identified with corresponding anti-rabbit IR-800 dye (1:30,000) and antimouse IR-680 dye (1:30,000) secondary antibodies incubation for 1 h at room temperature. Relative expression of specific immunoreactive band was calculated by comparing the fluorescence intensities in an Odyssey infrared imaging system (LI-COR) with respect to corresponding GAPDH.

### Luciferase reporter assay for promoter activity

Three micrograms per milliliter of *SLC44A4*, *SLC19A2*, and *SLC19A3* full-length as well as minimal promoter constructs (previously cloned and characterized in our laboratory; (45, 46, 47)) together with 100 ng of thymidine kinase promoter-Renilla luciferase plasmid (Promega) were transiently transfected into human colonic epithelial NCM460 cells utilizing Lipofectamine 2000 reagent (obtained from Life Technologies) for a period of 48 h, as described previously (45, 46, 47, 50). Cells were then exposed to chamber-induced hypoxic or normoxic conditions for additional 16 h and then lysed with passive lysis buffer for further analysis. All Renilla-normalized firefly luciferase promoter activities were assessed utilizing a Glomax 20/20 Luminometer (Promega) using the dual-luciferase assay system (Promega).

### Statistical analysis

All carrier-mediated TPP and thiamin uptake, qPCR, immunoblotting quantification, and luciferase reporter assay data were calculated as means ± standard error. The graphical data in all the figures were presented as percentage relative to simultaneously performed controls using GraphPad Prism 8 software (GraphPad Software, Inc). Statistical analyses were carried out by unpaired Student's *t* test, and *p* < 0.05 was considered as being statistically significant.

## Data availability

All data are contained within the article.

## Conflict of interest

The authors declare that they have no conflicts of interest with the contents of this article.
